# Acknowledgement to Reviewers of *Pathogens* in 2014

**DOI:** 10.3390/pathogens4010020

**Published:** 2015-01-08

**Authors:** Pathogens Editorial Office

**Affiliations:** MDPI AG, Klybeckstrasse 64, CH-4057 Basel, Switzerland

The editors of *Pathogens* would like to express their sincere gratitude to the following reviewers for assessing manuscripts in 2014:

Allen, Heather
Andere, Cecilia
Antonov, I
Baltrus, David
Van Françoise, Bambeke
Baron, Jody L.
Belser, Jessica
Benoit, Joshua
Berghman, Luc
Bisignano, Giuseppe
Borghi, Elisa
Bradford, B.J.
Campbell, Harry
Canani, Roberto
Canepari, Pietro
Carneiro, Victor Alves
Casey, John
Chauhan, Ashwini
Chen, Wangxue
Chinivasagam, Nalini
Choi, Eun-Kyoung
Chong, Parkson Lee-gau
Cirulli, Elizabeth T.
Clark, Clifford G.
Corthesy, Blaise
Daglia, Maria
Dai, Lei
Darkoh, Charles
Di Santo, James P.
Donkor, E. S.
Dorner, Marcus
Dwivedi, Hari P.
Engstrand, Lars
Esteve-Romero, Josep
Faleiro, Maria L.
Fallarero, Adyary
Fernandes, Elisabete
Foley, Steven
Fondi, Marco
Froelich, Brett
Galdiero, Stefania
Gillevet, Patrick
Gobet, Angélique
Goffard, Anne
Goldsmith, Jason
Grilli, Ester
Guerry, Patricia
Gumperz, J. E.
Haapalainen, Minna
Harmsen, Hermie J. M.
Hayashi, Tetsuya
Hennequin, Christiane
Heyndrickx, Marc
Hickey, Anthony J.
Hussain, Malik A.
Ibrahim, Salam A.
Ioos, Renaud
Iorio, Ronald M.
De Lucena, J. M. V. M.
Johnson, J. R.
Kelly, David J.
Kim, Jongheon
Kirby, Ralph
Knight, Rob
Köser, Claudio
Lawhon, Sara
Li, Zihai
Lobo-Castañón, Maria Jesus
Luke, Catherine
Lukšić, Ivana
Lynne, Aaron
Martinez, Jose Luis
Martinez-Urtaza, Jaime
Matthews, Karl R.
Minton, J. Ernest
Ramos-Morales, Francisco
Morton, Philip K.
Mountzouris, K. C.
Nachappa, Punya
Narayanavari, Suneel A.
Niemz, Angelika
Nilsson, R. Henrik
Norris, Steven
Oliver, James D.
Papatsiros, V. G.
Parker, Jennifer K.
Peeples, Mark
Perlin, David
Pettit, April C.
Piedra, Tony
Pierce, Ellen
Radicheva, Mariana
Romero, Ofelia
Rotger, Rafael
Sarris, Panagiotis F.
Schildgen, Oliver
Scholl, Peter F.
Scortichini, Marco
Sharma, Virag
Shukla, San Jay
Singhera, Gurpreet
Slana, I.
Smani, Younes
Snyder, Chris
Sokovic, Marina
Sokurenko, Evgeni V.
Stelinski, Lukasz L.
Tisa, Louis
Toju, Hirokazu
Tümmler, Burkhard
Ugorski, Maciej
Underwood, Anthony
Unnikrishnan, Meere
Valentin, Eulogio
Van Den Hoogen, Bernadette G.
Van Griensven, L. J. L. D.
Van Kaer, Luc
Vuillermin, Peter
Vuotto, Claudia
Waddell, Amanda
Walker, Lary C.
Welling, Mick
Wertz, Philip
Xie, Qingmei
Xu, Li
Yang, Liang
Yang, Zhaomin
Zeissig, Sebastian
Zhang, Liguo
Zhang, Qianyi
zhang, Wei
Zhu, Baoli
Zhu, Yefei

